# Racial Attitudes and Perceptions of Government Response during the COVID-19 Pandemic: Implications for Public Health Strategies

**DOI:** 10.3390/ijerph21091183

**Published:** 2024-09-05

**Authors:** Man Hung, Jeremy D. Franklin, William A. Smith, Carlos J. Crespo, Evelyn U. Ezikwelu, Jerry Bounsanga, Martin S. Lipsky

**Affiliations:** 1 College of Dental Medicine, Roseman University of Health Sciences, South Jordan, UT 84095, USA; 2 Huntsman Cancer Institute, Salt Lake City, UT 84112, USA; 3 Division of Public Health, University of Utah, Salt Lake City, UT 84108, USA; 4 College of Education, University of Utah, Salt Lake City, UT 84112, USA; 5 College of Science, University of Utah, Salt Lake City, UT 84112, USA; 6 University of Utah Health, University of Utah, Salt Lake City, UT 84132, USA; 7 Huntsman Mental Health Institute, Salt Lake City, UT 84108, USA; 8 College of Applied Health Sciences, University of Illinois Chicago, Chicago, IL 60612, USA; 9 Institute on Aging, Portland State University, Portland, OR 97201, USA

**Keywords:** racial inequality, politics, COVID-19, pandemic, economics, minorities, disparities

## Abstract

Background: This study explored whether opinions about the government’s role in addressing the COVID-19 pandemic vary based on demographic characteristics and racial beliefs. We hypothesized that opinions about the United States (U.S.) government’s response to COVID-19 would differ based on an individual’s characteristics such as age, race, and racial beliefs. Methods: We utilized an Inter-University Consortium for Political and Social Research dataset to examine differences in opinion regarding the government’s pandemic response, considering personal characteristics and racial beliefs. Descriptive statistics depicted respondents’ characteristics, and a Chi-square test for independence assessed whether differences emerged based on racial attitude, self-reported racial identity, sex, income, education, and age. Logistic regression analyses were conducted to independently determine which characteristics were associated with differences in evaluating the government’s pandemic response. Results: The sample consisted of 1028 respondents: 47.5% male and 52.5% female. Overall, the group viewed the government unfavorably, with only 40% reporting that the government responded correctly and 54% believing the government is almost always wasteful and inefficient. Hispanics or Latinos were more likely to view the government as wasteful or inefficient, while more Whites rated the government’s pandemic response as appropriate. Individuals who believed that racial discrimination is the main reason why many Black people cannot get ahead generally regarded the government’s pandemic response more favorably. Only 5% deemed the government’s response excessive. Being Black, younger, and female was associated with the view that racial discrimination is the main reason why many Black people cannot get ahead. Individuals who felt this way viewed the government unfavorably by almost a 2:1 ratio. Conclusions: A majority of U.S. residents do not believe the government responded correctly to the pandemic and more than half viewed the government as wasteful and inefficient. Differences emerged by ethnicity and racial attitudes, with individuals of color holding more negative views of the government’s response. Understanding this perspective can help develop messaging and strategies that resonate with communities where racial and minority groups live.

## 1. Introduction

The COVID-19 pandemic, triggered by the highly contagious severe acute respiratory syndrome coronavirus 2 (SARS-CoV-2) has swept the globe since early 2020. The Centers for Disease Control and Prevention identifies the key symptoms of COVID-19 as fever, cough, shortness of breath, fatigue, loss of taste or smell, and sore throat [1]. The impact of SARS-CoV-2 extends beyond health concerns, leading to significant economic and social upheaval, including rising unemployment, a decline in international trade, and widespread lockdowns. More than 700 million cases of COVID-19 and more than 7,000,000 deaths worldwide have been attributed to the disease and as of 20 April 2024 there were 111,820,082 cases and 1,219,487 deaths in the United States (U.S.) alone [2].

During the COVID-19 pandemic, governmental responses varied among countries. Outbreaks of known and new infections occur regularly [3] and despite warnings about the risks of a pandemic [4], the COVID-19 pandemic unmasked serious weaknesses in public health systems and the preparedness and efficacy of government responses to health crises. In Africa, nations were more vulnerable to COVID-19 because of insufficient financial investment in the health sector [5], while in Europe, even though governmental support was strong, messages critical of the government and conspiracy theories were still widely circulated [6]. Concerns about government reactions to health emergencies predate COVID-19. For example, systemic inequality and access to healthcare influenced public perceptions of government initiatives for AIDS, resulting in varying degrees of trust and compliance [7]. Previous epidemics such as the SARS, outbreaks of H5N1 flu, (2007), H1N1 (2009), Ebola virus, and the Zika epidemic in the Americas (2015–16) have shown that population compliance with government restrictions can make or break outbreak containment efforts [8]. A major determinant in how well citizens comply with government recommendations is trust [6].

In the United States, government took precautions to curb the spread of the virus, including travel bans [9] and passing the Coronavirus Aid, Relief, and Economic Security (CARES) Act to mitigate economic hardship [10]. The travel bans aimed to restrict entry into the U.S. from countries experiencing high infection rates. The CARES Act was a comprehensive legislative package designed to address the economic fallout from the pandemic. It included provisions for direct financial assistance to individuals, substantial aid to businesses, and significant allocations for healthcare providers. Additionally, the act included measures to bolster unemployment benefits, provide emergency funding to critically affected industries, and set aside funds for state and local governments grappling with sudden fiscal strain. These measures aimed to provide immediate relief to individuals and businesses and set the groundwork for a more resilient economic recovery.

However, racially minoritized groups argued that the CARES Act stimulus was insufficient for minorities who faced systemic inequalities before the pandemic [11]. Before the pandemic, 22% of African Americans and 19% of Hispanics fell below the poverty line, compared to 9% for Whites [12]. These disparities contributed to COVID-19, disproportionately affecting the elderly, low-income, marginalized, and other vulnerable populations. It is not surprising that financial and social constraints made it challenging for marginalized racial and ethnic groups to follow mitigation strategies [13]. Some suggest that social distancing mandates worsened the economic situations of racially minoritized families [14] and that inadequate governmental responses widened existing pre-pandemic racial, financial, and health disparities [15].

Socio-political ideologies shape individual perspectives on the COVID-19 pandemic and the accompanying societal changes. Studies indicate a noticeable divergence in attitudes based on these ideologies. Individuals who self-identify as liberal or moderate tend to exhibit heightened concern regarding the pandemic and are generally more supportive of implementing societal restrictions, such as lockdowns and mandatory mask-wearing, to curb the spread of the virus [16,17]. In contrast, those who identify as conservatives are often less inclined to perceive COVID-19 as a severe threat and may exhibit less support for stringent public health measures.

Moreover, the intersection of socio-political ideologies with experiences of racial inequality remains an under-explored area in pandemic research. Given the significant impact of racial disparities observed during the pandemic, from infection and mortality rates to economic consequences, understanding how these disparities intersect with political beliefs is vital. This intersection could significantly influence perceptions of government responses to the pandemic. Communities disproportionately affected by COVID-19 may view government strategies through the lens of existing inequalities, potentially affecting their trust and compliance with public health directives. Such insights are crucial for developing more effective, inclusive pandemic-related strategies that acknowledge and address the diverse experiences and viewpoints of the population. Systemic racism also contributes to greater distrust among marginalized groups [18].

Public trust in governmental institutions influences adherence to a pandemic response [6,18]. Trust for healthcare institutions is eroding in the U.S. [19] and institutional distrust among minorities exacerbates COVID-19 inequities [20]. Studies that examine how racial inequalities and socio-political ideologies influence views on governmental pandemic responses could provide valuable information to guide more effective communication strategies, policy-making, and public health interventions that resonate across the diverse spectrum of the population. This offers opportunities for ensuring equitable and effective management of current and future public health crises. This study explored opinions about the government’s role in addressing economic hardship incurred by the COVID-19 pandemic with a focus on how these views might differ by race and personal characteristics. Understanding attitudes toward government responses can inform policymakers about strategies to address public interests, manage economic needs, and understand the impact of COVID-19 on racially marginalized members of society. Such insights are crucial for developing more effective, inclusive pandemic-related strategies that acknowledge and address the diverse experiences and viewpoints of the population.

## 2. Methods

### 2.1. Data Source

We utilized data from the Inter-university Consortium for Political and Social Research (ICPSR) database, specifically the study titled “*Political and Personal Reactions to COVID-19 During the Initial Week of Social Distancing in the United States*” [21]. The consortium collected cross-sectional survey data from 1030 respondents aged 18 years and over across the United States. This sample was developed to be demographically representative using census-derived data on sex, race, and income [22]. Data were collected from 3 March 2020, to 31 March 2020. The survey included sociodemographic information and assessed personal attitudes related to COVID-19, as well as perceptions of the government’s response to alleviate economic suffering, categorized by political beliefs and sociodemographic factors. More detailed information about the survey can be found at https://doi.org/10.3886/E119629V1 (accessed on 6 May 2024).

### 2.2. Measures

This study focused on individuals’ beliefs about the government’s role in alleviating economic hardship for people of all races during the COVID-19 pandemic. The key study variables included “PolGov,” which measured an individual’s political opinion about the government, with responses coded as 1 for “Government is almost always wasteful and inefficient” and 2 for “Government often does a better job than people give it credit for”. Another key variable was “GovResp”, which assessed an individual’s opinion about the government’s responses to COVID-19, coded as 1 for “Not done enough in response to COVID-19”, 2 for “Responded correctly to COVID-19”, and 3 for “Done too much in response to COVID-19”. Additionally, the variable “PolRace” measured which of two sentiments regarding race most closely aligned with the respondent’s belief, coded as 1 for “Blacks who can’t get ahead in this country are mostly responsible for their own condition” and 2 for “Racial discrimination is the main reason why many Black people can’t get ahead these days”.

The respondents’ demographics were included as control variables because they can influence individuals’ personal and political ideologies. Specifically, the analyses controlled for age, sex (male, female), ethnicity (Hispanic or Latino, not Hispanic or Latino), and race (White, Black or African American, Asian, American Indian or Alaska Native, Native Hawaiian or Pacific Islander). Additionally, the analysis controlled for three indicators of socioeconomic status: educational level, income, and marital status.

Educational level was categorized into seven levels: 1 for less than a high school degree, 2 for high school graduate (high school diploma or equivalent, including GED), 3 for some college but no degree, 4 for an associate degree in college (2-year), 5 for a bachelor’s degree in college (4-year), 6 for a master’s degree, and 7 for an advanced degree (JD, PhD, MD, etc.). Income was grouped into four categories: less than USD 40,000, USD 40,000 to USD 79,000, USD 80,000 to USD 100,000, and more than USD 100,000. Marital status was divided into three levels: 1 for married, 2 for formerly married (widowed/divorced/separated), and 3 for never married. Table 1 summarizes the demographic distribution of the study sample.

### 2.3. Statistical Analysis

Descriptive statistics, including frequencies, percentages, and means, were used to describe the respondents’ age, sex, income, education level, ethnicity, and racial identities. The Chi-square test for independence was employed to determine whether political ideologies differed significantly by racial attitudes (PolRace) and to test for significant differences between respondents’ personal and political ideologies about government, governmental responsibilities, and racial identities. Bivariate analyses were conducted to examine the differences between PolRace and demographic characteristics.

Logistic regression analyses were performed to independently examine the associations of PolGov and GovRes with PolRace, controlling for significant demographic characteristics. All statistical tests were conducted using SPSS version 29, and significance was set at an alpha level of 0.05.

## 3. Results

The nationally representative sample comprised 1030 respondents. The demographic details are presented in Table 1. The gender distribution was fairly balanced, with males representing 47.5% and females 52.5%. Ethnically, the respondents were diverse: 11.9% identified as Hispanic or Latino, 53.6% as White, 23.1% as Black or African American, and 11.6% as Asian. Those identifying as American Indian or Alaska Native, Native Hawaiian, or Pacific Islander comprised 2.7%. Additionally, 5.4% categorized themselves as ‘Other’, and 3.6% reported mixed ethnicity.

Regarding income levels, about 7% of respondents reported a pretax income below USD 10,000 annually, while 7.4% reported an income exceeding USD 150,000. In terms of educational attainment, 20% of the participants reported completing high school as their highest level of education, and 16% held advanced degrees, including master’s, JD, PhD, MD, and other similar qualifications. The majority of respondents were married.

Table 2, Table 3, Table 4 and Table 5 summarize the demographic breakdown by political question responses. There was no significant difference in the respondents’ age among the types of government responses reflecting opinions (Table 2). A larger proportion of Hispanic/Latino respondents (60.5%) than non-Hispanic/Latino respondents (52.25%) believed that the government is wasteful and inefficient (*p* = 0.008) (Table 2). On average, older individuals (mean = 51.81 years) believed that Blacks who cannot get ahead in this country are mostly responsible for their condition, whereas younger individuals (mean = 46.09 years) believed that it is caused by racial discrimination (*p* < 0.0001) (Table 3). More males (52.76%) believed that Blacks who cannot get ahead in this country are mostly responsible for their condition, whereas more females (56.38%) believed that Blacks could not get ahead due to racial discrimination (*p* = 0.003) (Table 3). Almost 60% of females (compared to 50.10% of males) indicated that the government had not done enough in response to COVID-19 (*p* = 0.006) (Table 4).

Approximately 5% of individuals felt the government’s pandemic response was excessive (Table 5). More people (64.15%) who believed that Blacks could not get ahead due to racial discrimination also believed that the government response to COVID-19 was insufficient (Table 6). After adjusting for demographic characteristics, those who felt Blacks could not get ahead due to racial discrimination exhibited 92% greater odds of believing the government has not done enough rather than doing too much in responding to COVID-19 (Odds Ratio = 1.920; *p* = 0.033) (Table 7).

## 4. Discussion

Trust in government is built and maintained by several factors. In this study, we explored the relationship between race, racial attitudes, and perceptions of the U.S. government’s handling of the COVID-19 pandemic. Our findings provide crucial insights into the dynamics of political trust and public health, particularly in a racially diverse society. We discovered several significant insights. First, a majority (54%) of the participants deemed the government’s response as largely inefficient and wasteful, while only 40% believed it was handled correctly. Governmental actions can influence the course of pandemics and these concerns may be one reason that despite being one of the richest countries, the U.S. ranks among the leaders in COVID-19 cases and deaths [23]. 

The finding that more respondents perceive the government’s response as inefficient highlights a significant trust deficit, which may have broader implications for public health policy and compliance. This is particularly concerning as lower levels of trust can lead to reduced adherence to public health guidelines, thereby exacerbating the impact of the pandemic. Notably, opinions varied significantly across different ethnic and racial groups. Hispanic or Latino participants were more inclined to perceive the government as inefficient or wasteful. Moreover, the approval of the government’s response to the pandemic varied with race; White participants were more likely to approve of the government’s actions compared to other races. These findings align with Abramson’s political reality model, formulated in 1980, which suggests that Black individuals tend to have less political trust than Whites, partly due to historical exclusion from political power [24]. This framework remains relevant, as recent studies support the idea [25,26,27]. Our results, indicating that minority groups view the government’s pandemic response as less effective, underscore a persistent divide in political trust between Black and White individuals. A lack of confidence influences beliefs and actions [28] that can undermine an individual’s adherence to public health policy. In contrast, increases in political trust are associated with significant decreases in infected cases and COVID-related deaths [29].

This racial divide in trust has important implications for public health strategy. It suggests that interventions designed to increase trust among minority communities could be pivotal in improving public health outcomes, particularly in managing pandemic responses. Second, differences in racial attitudes significantly influenced perceptions about the government’s pandemic response. Understanding whether people perceive the government as doing too little or too much is a key factor affecting trust in government [30]. Individuals who believed that racial discrimination is the main reason many Black people cannot get ahead generally regarded the government’s pandemic response as insufficient. Fewer individuals thought the government responded adequately, and only 5% deemed the government’s response excessive. After controlling for other variables, individuals who felt racial discrimination was the main reason many Black people cannot get ahead viewed the government unfavorably by almost a 2:1 ratio. Our study also found that being Black, younger, and female was associated with the view that racial discrimination is the main reason many Black people cannot get ahead, a result consistent with other reports [31].

The significant association between beliefs about racial discrimination and views on the government’s pandemic response highlights the deep-seated influence of societal attitudes on political trust. This finding suggests that addressing racial discrimination is not only a matter of social justice but also critical for enhancing public health policy effectiveness. If minority groups perceive government actions through the lens of discrimination, they are less likely to trust and comply with those actions, which can have serious consequences for public health.

It should not be surprising that views about the government’s pandemic response link to beliefs about racial discrimination. Societal attitudes influence the beliefs and attitudes people endorse, and Blacks and Latinos expect racial bias when White governmental officials make decisions [32,33]. Societal attitudes about race, whether they involve unconscious bias, stereotyping, or prejudice, can also contribute to healthcare disparities [34]. A preponderance of evidence suggests that racial and ethnic minority groups are disproportionately affected by COVID-19 and experience a greater risk of infection, hospitalization, and death [35]. These same groups are disproportionately affected by underlying medical conditions such as cardiovascular disease, chronic kidney disease, chronic obstructive pulmonary disease, diabetes, hypertension, human immunodeficiency virus, and obesity [36]. However, while Gollust et al. [37]. found that respondents to a nationally representative survey recognized age and underlying health conditions as risk factors for COVID-19 morbidity, fewer recognized the disproportionate effect of social group disparities. Only about half recognized that individuals from lower socioeconomic status are more likely to die from COVID-19 than wealthier people or that Black people experience more COVID-related disparities compared to White people.

The persistent health disparities among racial minorities, even after accounting for socioeconomic factors, underscore the role of structural racism in shaping health outcomes. Our study’s finding that beliefs about racial discrimination strongly influence perceptions of government response suggests that public health strategies must consider the broader social context in which these beliefs are formed. While social group disparities can be attributed to socioeconomic factors, health disparities persist in minority communities even after accounting for socioeconomic factors, suggesting structural racism is a contributing factor. Researchers argue that rates of morbidity, mortality, and overall well-being depend on socially assigned race [38] and that biological risk factors for COVID-19 like diabetes, obesity, asthma, and hypertension can reflect sociological influences [39]. Therefore, it is not surprising that after controlling for socioeconomic factors, our study still found that those who believe racial discrimination inhibits advancement emerged as a decisive factor for the belief that the government is not doing enough to address the pandemic. These views are likely reinforced by a widening gap of economic disparities among racial minorities attributed to the COVID-19 pandemic.

### 4.1. Implications

The finding that those who acknowledge the role of racism in societal advancement are more critical of the government’s pandemic response underscores the necessity for a deeper understanding of how best to support minority communities. This suggests that standard public health messages may not be effective across all communities, particularly among those that have historically been marginalized. Tailoring public health messages to address the specific concerns and contexts of minority communities could significantly improve trust and compliance, leading to better health outcomes. This is especially relevant in crafting public health messaging which can affect the impact of a public health crisis. For instance, standard fact-based communication about social distancing might not resonate effectively in densely populated areas or with those who cannot work from home and are required to be physically present at their jobs.

It is crucial to recognize that political and racial identities vary significantly among individuals of color; as such, no single pandemic response strategy can address all disparities. Despite this, acknowledging that racial attitudes significantly influence opinions about the pandemic response can be instrumental in developing more effective prevention and intervention strategies. A nuanced approach that considers these differences is vital. Public health strategies must move beyond a one-size-fits-all approach and instead develop targeted interventions that address the specific needs and concerns of different racial and ethnic groups. An important strategy in this regard is the formation of broad coalitions that include community representatives and leaders. Coalitions are better positioned to tailor governmental responses to the specific needs and contexts of communities where racial and minority groups are situated. By involving those who live, learn, work, play, and worship in these communities, responses can be more appropriately adapted to their unique circumstances and challenges. This approach not only ensures that the interventions are more relevant and effective but also fosters a sense of involvement and ownership among the communities, potentially enhancing the acceptance and public trust of public health initiatives. Higher levels of trust in government trust have been linked to lower infection and fatality rates during the COVID-19 pandemic [40], highlighting the critical importance of strengthening public confidence in government agencies.

### 4.2. Strengths and Limitations

This study, while insightful, has certain limitations. Primarily, it relies on cross-sectional data, which captures a specific moment in time. The perspectives and attitudes reflected in this study may shift as time progresses. Nonetheless, this study offers a crucial message to policymakers: racial views significantly influence reactions to government decisions. This finding is particularly relevant for ongoing and future public health crises, where trust in government will be a key determinant of the success of public health measures. Understanding the role of racial attitudes can help in designing more effective, equitable, and inclusive public health policies. Another limitation concerns the representativeness of the sample. Although designed to mirror national demographics, there might be subtle, unmeasurable differences from the broader U.S. population. For example, households with children were under-represented in our sample (28.9%) compared to their actual proportion in the U.S. households (45.0%).

Furthermore, the survey design, which often relied on binary choices like yes/no for complex issues, may have constrained the respondents’ ability to express their views fully. This limitation highlights the need for future research to adopt more nuanced survey instruments that can capture the complexity of opinions, particularly on issues as multifaceted as racial attitudes and trust in government. It is particularly pertinent for multifaceted topics such as racial attitudes and perceptions of government efficiency and response, where nuanced opinions are likely to be prevalent. Additionally, the timing of the data collection, during an election year in a politically charged environment, could have influenced the responses. The study also did not differentiate between attitudes toward different levels of government (state, local, federal), which might have varying influences on the respondents’ opinions.

Future research should aim to delve deeper into the reasons behind these views to address disparities in public health better. It would also be beneficial to explore how social distancing and other pandemic-related measures are perceived and practiced among different racial/ethnic groups, political affiliations, and across various government agencies. Such detailed analyses would provide a more comprehensive understanding of the complex dynamics and inform more targeted and effective policy responses.

### 4.3. Conclusions

This study provides valuable insights into the complex relationships between race, racial attitudes, and perceptions of the government’s handling of the COVID-19 pandemic. Our findings indicate that a significant portion of the population, particularly among minority groups, views the government’s response as inefficient and insufficient. These perceptions are deeply intertwined with broader issues of political trust and racial discrimination, which have historically shaped the experiences and attitudes of racial and ethnic minorities in the U.S. The findings underscore the need for public health strategies that are tailored to the diverse experiences of racial and ethnic communities. A one-size-fits-all approach is insufficient; instead, targeted interventions that address specific concerns and build trust within these communities are essential for effective public health outcomes.

## Figures and Tables

**Table 1:** Demographic characteristics (N = 1030).

Variable	Mean (SD)	Min/Max	n	%
Age (in years)	48.83 (18.73)	18/80+	1030	100
Sex				
Male			489	47.5
Female			541	52.5
Ethnicity *				
Hispanic or Latino			123	12.2
Non-Hispanic or Latino			889	87.8
Race				
White			552	53.6
Black or African American			238	23.1
Asian			119	11.6
American Indican or Alaska Native or Native Hawaiian or Pacific Islander			28	2.7
Other			56	5.4
Mixed			37	3.6
Marital status				
Married			447	43.4
Formerly Married (widowed/divorced/separated)			206	20.0
Never Married			377	36.6
Education				
High school graduate or less than high school degree			208	20.2
Some college but no degree			243	23.6
Associate degree in college (2-year)			135	13.1
Bachelor’s degree in college (4-year)			279	27.1
Advanced degree (master’s, JD, PhD, MD, etc.)			165	16.0
Income				
Less than USD 40,000			409	41.7
USD 40,000 to USD 79,999			344	33.4
USD 80,000 to USD 99,999			93	9.0
USD 100,000 or more			184	17.9

Note: * = 1.75% missing data.

**Table 2:** Demographics of PolGov * responses.

Characteristic	Total	PolGov	PolGov	*p*-Value
	Sample	Wasteful/Inefficient	Better than	Sample
	N = 1030	n = 556	n = 473	
Age (in years)	48.83	48.47	49.21	0.530
Sex				0.549
Male	489	269	220	
Female	540	287	253	
Ethnicity				0.008
Hispanic or Latino	123	80	43	
Not Hispanic or Latino	888	464	424	
Race				0.421
White	551	292	259	
Black or African American	238	128	110	
Asian	119	61	58	
American Indian or Alaska				
Native or Native Hawaiian				
Or Pacific Islander	28	15	13	
Other	56	35	21	
Mixed	37	25	12	

Note: * Survey item: Government is almost always wasteful and inefficient or better than credited.

**Table 3:** Demographics of PolRace * responses.

Characteristic	Total	PolRace	PolRace	*p*-Value
	Sample	Mostly Responsible for Their Own	Racial Discrimination Main Reason	
	N = 1030	n = 494	n = 536	
Age (in years)	48.83	51.81	46.09	<0.001
Sex				0.003
Male	489	258	231	
Female	541	236	305	
Ethnicity				0.224
Hispanic or Latino	123	53	70	
Not Hispanic or Latino	889	435	454	
Race				<0.001
White	552	313	239	
Black or African American	238	73	165	
Asian	119	59	60	
American Indian or Alaska				
Native or Native Hawaiian				
Or Pacific Islander	28	14	14	
Other	56	22	34	
Mixed	37	13	24	

Note: * Survey item: Blacks who can’t get ahead in this country are mostly responsible for their own condition, or Racial discrimination is the main reason why many black people can’t get ahead these days.

**Table 4:** Demographics by GovResp * responses.

Characteristic	Total	PolResp	PolResp	PolResp	*p*-Value
	Sample	Not Done Enough	Responded Correctly	Done Too Much	
	N = 1030	n = 569	n = 410	n = 51	
Age (in years)	48.83	48.36	50.55	40.43	<0.001
Sex					0.006
Male	489	245	215	29	
Female	541	324	195	22	
Ethnicity					0.695
Hispanic or Latino	123	68	47	8	
Not Hispanic or Latino	889	487	359	43	
Race					<0.001
White	552	267	257	28	
Black or African American	238	150	74	14	
Asian	119	78	37	4	
American Indian or Alaska					
Native or Native Hawaiian					
Or Pacific Islander	28	10	17	1	
Other	56	39	16	1	
Mixed	37	25	9	3	

Note: * Survey item about government: Not done enough in response to COVID-19; responded correctly to COVID-19; or done too much in response to COVID-19.

**Table 5:** Description of personal ideologies.

Variable	N (%)	*p*-Value
PolGov		<0.001
Government is almost always wasteful and inefficient	556 (54.1)	
Government often does a better job than people give it credit for	473 (45.9)	
GovResp		<0.001
Government has not done enough in response to COVD-19	569 (55.2)	
Government responded correctly to COVID-19	419 (39.8)	
Government has done too much in response to COVID-19	51 (5.0)	
PolRace		<0.001
Blacks who can’t get ahead in this country are mostly		
Responsible for their own condition	494 (48.0)	
Racial discrimination is the main reason why many black		
People can’t get ahead these days	536 (52.0)	

**Table 6:** PolGov and GovResp by PolRace.

Variable	PolRace	PolRace	*p*-Value
	Mostly Responsible for Their Own	Racial Discrimination Main Reason	
	n (%) *	n (%) *	
PolGov			
Wasteful/inefficient	286 (55.44%)	279 (48.56%)	0.017
Better than credited	208 (43.97%)	265 (56.03%)	
GovResp			
Not done enough	204 (35.85%)	365 (64.15%)	<0.001
Responded correctly	265 (64.63%)	145 (43.37%)	
Done too much	25 (49.02%)	26 (50.98%)	

Note: * May not add up to 100 because of rounding.

**Table 7:** Logistic regression analyses related to PolRace (controlling for demographic variables—age, sex, and race).

Variable	Odds Ratio	95% CI	*p*-Value
PolGov			
Wasteful/inefficient	1.830	[1.390, 2.410]	<0.001
Better than credited (reference)			
GovResp			
Not done enough	1.920	[1.053, 3.502]	0.033
Responded correctly	0.538	[0.292, 0.993]	0.047
Done too much (reference)			

## Data Availability

Data are freely available at https://doi.org/10.3886/E119629V1 (accessed on 6 May 2024).
